# How best to structure interdisciplinary primary care teams: the study protocol for a systematic review with narrative framework synthesis

**DOI:** 10.1186/s13643-016-0339-9

**Published:** 2016-10-04

**Authors:** W. Dominika Wranik, Jill A. Hayden, Sheri Price, Robin M.N. Parker, Susan M. Haydt, Jeanette M. Edwards, Esther Suter, Alan Katz, Liesl L. Gambold, Adrian R. Levy

**Affiliations:** 1 School of Public Administration, Faculty of Management, Dalhousie University, Halifax, Canada; 2 Department of Community Health and Epidemiology, Faculty of Medicine, Dalhousie University, Halifax, Canada; 3 Faculty of Nursing, Dalhousie University, Halifax, Canada; 4 Kellog Library, Dalhousie University, Halifax, Canada; 5 Faculty of Management, Dalhousie University, Halifax, Canada; 6 Primary Health Care and Chronic Disease, Winnipeg Regional Health Authority, Winnipeg, Canada; 7 Workforce Research and Evaluation, Alberta Health Services, Calgary, Canada; 8 Department of Community Health Sciences, Faculty of Health Sciences, University of Manitoba, Winnipeg, Canada; 9 Department of Sociology and Social Anthropology, Faculty of Arts and Social Sciences, Dalhousie University, Halifax, Canada; 10 Department of Community Health and Epidemiology, Faculty of Medicine, Dalhousie University, Halifax, Canada

## Abstract

**Background:**

Western publicly funded health care systems increasingly rely on interdisciplinary teams to support primary care delivery and management of chronic conditions. This knowledge synthesis focuses on what is known in the academic and grey literature about optimal structural characteristics of teams. Its goal is to assess which factors contribute to the effective functioning of interdisciplinary primary care teams and improved health system outcomes, with specific focus on (i) team structure contribution to team process, (ii) team process contribution to primary care goals, and (iii) team structure contribution to primary care goals.

**Methods and design:**

The systematic search of academic literature focuses on four chronic conditions and co-morbidities. Within this scope, qualitative and quantitative studies that assess the effects of team characteristics (funding, governance, organization) on care process and patient outcomes will be searched. Electronic databases (Ovid MEDLINE, Embase, CINAHL, PAIS, Web of Science) will be searched systematically. Online web-based searches will be supported by the Grey Matters Tool. Studies will be included, if they report on interdisciplinary primary care in publicly funded Western health systems, and address the relationships between team structure, process, and/or patient outcomes. Studies will be selected in a three-stage screening process (title/abstract/full text) by two independent reviewers in each stage. Study quality will be assessed using the Mixed Methods Assessment Tool. An a priori framework will be applied to data extraction, and a narrative framework approach is used for the synthesis.

**Discussion:**

Using an integrated knowledge translation approach, an electronic decision support tool will be developed for decision makers. It will be searchable along two axes of inquiry: (i) what primary care goals are supported by specific team characteristics and (ii) how should teams be structured to support specific primary care goals? The results of this evidence review will contribute directly to the design of interdisciplinary primary care teams. The optimized design will support the goals of primary care, contributing to the improved health of populations.

**Systematic review registration:**

PROSPERO CRD42016041884

**Electronic supplementary material:**

The online version of this article (doi:10.1186/s13643-016-0339-9) contains supplementary material, which is available to authorized users.

## Background

Mature health systems are placing increased emphasis on interdisciplinary teams to deliver primary care [1, 2]. Interdisciplinary primary care (IDPC) teams consist of healthcare providers from different disciplines working together toward common goals [2]. This approach is perceived as appropriate to address health needs of populations through the creation of comprehensive care options, increased continuity, and coordination of care [3–7].

The policy maker perspective is captured in Hutchison et al. [8]. Provinces in Canada have been implementing IDPC teams and “this is an opportune time for within and cross-jurisdictional comparisons” and to “understand the complicating effects of physician remuneration and the variety of organizational forms” (p. 281). Now more than ever do we need a synthesis of the evidence, both to guide future implementations and to improve existing teams [8].

In addition to decision makers, providers crave evidence. Providers may be reluctant to organize into new teams without evidentiary support [9]. For example, physicians in Ontario have been seeking evidence regarding the lessons learned and best practices of Ontario’s Family Health Teams [10]. The Knowledge User Advisory Group associated with this project has identified the question of how best to structure and support IDPC teams as being of utmost importance. This is consistent with findings from a Research Roundtable that was held in October 2014 as a part of a qualitative study of the funding and remuneration structures of IDPC teams in Canada [11].

The ongoing request for more evidence makes this synthesis of such evidence across Canada and similar health systems timely. Previous evidence syntheses about IDPC teams have looked at specific clinical treatments [12–14] or isolated particular variables with respect to team functioning [15–17]. These reviews have been driven by the quantitative method, which tend to ignore the complexity of context. Their results are of limited usefulness to decision makers. Our study will improve upon existing literature via its comprehensive focus and rigorous inclusion and evaluation of qualitative alongside quantitative evidence [18]. Our results will explicate what is known about the implications of varied choices in varied contexts.

This knowledge synthesis will deliver a decision support tool to knowledge users, some of whom are members of the Knowledge User Advisory Group working with this research team. The question of how best to structure IDPC teams has been at the forefront of their policy agenda. They have committed to using this tool in policy and planning discussions. The tool will support evidence-based policy development and implementation strategies in the organization of primary care. Well-organized teams improve primary care delivery [8, 19], and an improved primary care system is shown to positively contribute to improved population health [20].

This knowledge synthesis builds on three prior projects: (i) a preliminary scoping of relevant literature [21], (ii) a qualitative study of IDPC teams in Canada [22], and (iii) a systematic review with narrative synthesis focused on the impact of funding and remuneration on team process. The focused review allowed us to pilot the search and selection approach. The qualitative study and scoping review allowed us to identify relevant team characteristics, categorize them (financial, governance, management), identify measures of team process, and identify relevant health system outcomes [3–7, 23–34].

### Objectives

The goal of this evidence synthesis is to review the relevant published literature to identify factors that contribute to the effective functioning of IDPC teams and improved health system outcomes. Specific objectives are as follows:

1. To assess the extent to which *team structure* contributes to *team processes*
2. To assess the extent to which *team processes* contribute to *primary care goals*
3. To assess the extent to which *team structure* contributes to *primary care goals*

We will undertake a synthesis of the research evidence to help answer decision makers’ question: “Given my goal and context, how best to structure IDPC teams?”

Based on a scoping review and a qualitative study of IDPC teams in Canada (under review), we define the following indicators to measure the relevant factors:

*Team structure*: We categorize factors related to team structure into financial structure (funding method for team, remuneration method for providers), governance structure (lines of accountability, type of governance), and management structure (team composition, management of patient acuity, management of team, location, patient rostering, and list size).

*Team processes*: This includes factors related to the functioning of teams, including the Team Climate Inventory [24, 35] and the Team Effectiveness Tool [25].

*Primary care goals*: These are relevant health system outcomes including patient health or process outcomes specific to selected chronic conditions (described below) and primary care delivery process indicators including access, comprehensiveness, and continuity, or broader measures, such as the primary care assessment tool [36].

## Methods

### Systematic literature search

Following the PRISMA reporting guidelines [37] and the Cochrane Handbook [38] in a context that is not strictly clinical [39], this review will follow six key steps: (1) defining the review question, (2) identifying studies, (3) selecting studies, (4) critical appraisal of studies, (5) collecting data, and (6) synthesizing and interpreting results. We also add (7) knowledge translation. A PRISMA-P checklist is included in the Additional file 1.

#### Defining the review questions

Our research question, refined with input from the Knowledge User Advisory Group, is *Which factors* (*team structure; team process*) *positively contribute to the functioning of interdisciplinary primary care teams and support primary care goals?*

#### Identifying studies for review

We will search for and synthesize two types of evidence:

- Peer-reviewed academic literature as found through a systematic search, including reference searches
- Grey literature, including non-peer-reviewed studies and online reports found through a structured online web search

##### Systematic search of academic literature

1. A comprehensive electronic search will be conducted with the help of a library scientist (RP) using indexed and free text words, with no date limitations, in the following databases: Ovid MEDLINE, Embase, CINAHL, and PAIS. Our search strategy will include the following key terms (and synonyms): primary care, interdisciplinary teams, interprofessional practice, organization, structure, composition, team size, providers, patient list size, physician leadership, fee-for-service, colocation, team process, team climate inventory, and team effectiveness. Consult Additional file 2 for the comprehensive search strategy in MEDLINE.
2. The primary search of academic databases is focused on four specific conditions: diabetes, asthma, ischemic heart disease, and hypertension, as well as general chronic disease management.
3. Reference searches of relevant reviews will be conducted, including [12–17] and other reviews found through the focused electronic search.
4. Citation searches of specific seminal/older studies [23, 33, 40].

##### Structured search of non-academic documents

The grey literature search will be guided by the Grey Matters search tool produced by the Canadian Agency for Drugs and Technologies in Health (CADTH). The tool provides a list of websites as related to specific subject areas and a system for tracking the searches. We modify the list of websites, because the Grey Matters is focused on clinical interventions rather than health services research. Additional file 3 includes the full list of websites to be searched. All searches will be tracked using the Grey Matters system. Key terms will be adapted from the academic literature search.

#### Selecting studies for inclusion

Studies will be selected for inclusion using a three-phase process of title screening, title/abstract screening, and full text screening/review. Inclusion is generous in terms of methodology. Our scoping review revealed that most studies are qualitative, and many of the quantitative studies have a non-experimental research design. We will include studies assessed as having strong and weak evidence (ranked using the Mixed Methods Assessment Tool, MMAT, described below). The purpose of the review is to support decision-making, which includes a clear identification of those parts of policy discussions for which evidence is weak, in addition to the identification of strong evidence.

##### Inclusion and exclusion criteria

Inclusion criteria were developed on the basis of a scoping review, a qualitative study, and consultations with the Knowledge User Advisory Group and the research team. To be included, studies must report data on interdisciplinary primary care in publicly funded systems in Western health systems. They must address the relationships between team structure and team process, or between team structure and health system outcomes, or between team process and health system outcome. Studies focused on four key areas of primary care will be included: asthma, diabetes, hypertension, and ischemic heart disease. Only primary studies will be included. Opinions, editorials, and other studies with no primary data will be excluded. Our focus is on studies in the English language. French language articles discussing Western health systems will be assessed, as we have working knowledge of the French language.

##### Title screening

Two researchers will independently review the titles of all articles with the purpose of discarding obviously irrelevant titles (i.e., those not related to primary care teams). In case of any ambiguity, including disagreement between reviewers, the study will advance to the next screening level.

##### Title/Abstract screening

Two researchers will independently review the titles/abstracts advanced from the previous stage. Abstracts for which there is disagreement between the two initial reviewers will be reviewed by a third researcher (the principal investigator) with discussion to reach consensus. Studies will advance, if they appear potentially relevant but the abstract is not sufficiently detailed (e.g., how is the structure of the team captured).

##### Full article screening/review

Two researchers will independently review full text articles to assess all inclusion criteria. Discrepancies will be discussed. Remaining discrepancies will be reviewed by a third researcher (the principal investigator) with the pivotal vote. The final step in the study selection is also the first step of the qualitative analysis.

#### Data extraction and analysis of studies

Based on the preliminary scoping of the literature, we expect to find primarily qualitative studies and a smaller number of quantitative studies. This calls for a mixed studies review [41]. We will take an integrated approach, where quantitative studies are qualified, then all studies are subjected to qualitative synthesis [18, 40, 41], also referred to as a convergent design [41]. This is appropriate given the diverse nature of the quantitative studies (variety of contexts, team characteristics, outcome measures) [42].

Data extraction facilitates the analysis (decomposition into parts) of included studies. Given that the purpose of our synthesis is to provide evidence for decision support [43], analysis will be conducted using a framework approach, which offers a structured approach to organizing and analyzing large volumes of information resulting from qualitative research [44, 45]. The approach facilitates the handling of large volumes of information from qualitative research by using an a priori framework informed by background research and research team discussions. The framework is also used to develop the decision-support tool (Additional file 4: Table S1).

We will extract information on the characteristics of included studies, such as care setting (e.g., region, rural or urban, general or focused care), patient population (general or specialized), research question, study design, type of data, analysis, results, and conclusions [46]. We will extract information about the structural characteristic described (policies and procedures, team composition, provider remuneration, team funding, team governance) and the care process or outcome to which it is linked (team process, health services process, diabetes care, hypertension care, asthma care, ischemic heart disease care, or other chronic disease management outcomes). In line with the integrated approach to mixed studies reviews, we will qualify quantitative studies [42, 43, 47, 48]. This is one type of what is also referred to as Bayesian conversion and specifically means that all quantitative data are thematically synthesized and codified according to the strength of the effect [18]. Data will be extracted by two researchers independently, and discrepancies will be resolved as described under “full title screening.”

Our analytical framework is presented in tabular format (Additional file 4: Table S1). It is also the structure of the online decision aid tool. The framework will be populated by the description of existing evidence, the quality rating of the evidence (see below), and the absence of evidence. For example, the shaded cell in the table will contain information about what is known about the relationships between team size and comprehensiveness of care. The framework will be modified to accommodate emergent categories, as needed.

#### Critical appraisal of studies

All studies will be critically assessed for quality using the Mixed Methods Appraisal Tool which allows to rate each qualitative and quantitative study along a four-point scale [49, 50] by two independent reviewers. In case of disagreement between reviewers, consensus will be reached via discussion and consultation with a third reviewer. The reviewers will not be blinded to authors, institution, or journal of publication due to feasibility [38].

The Mixed Methods Appraisal Tool (MMAT) is specifically designed for the appraisal stage of complex systematic literature reviews that include qualitative, quantitative, and mixed methods studies (mixed studies reviews). The MMAT was designed based on the examination of existing reviews, and the literature speaking to criteria for planning, designing, and reporting mixed methods research. [50, 51]

The MMAT permits to concomitantly appraise, describe, and score the methodological quality for three methodological domains: mixed, qualitative, and quantitative (subdivided into randomized controlled, non-randomized, and descriptive). For each relevant study selected, the methodological quality can then be described using the corresponding criteria. For each study, an overall quality score is calculated along a four-point scale (* one criterion met to **** all criteria met). Mixed methods studies are ranked by their weakest component.

In the case of poor reporting, study authors will be contacted for additional information, and/or corresponding publications will be consulted. This is considered standard practice when reviewing qualitative studies. [50, 52]

#### Synthesis and interpretation of results

Our review will include a narrative synthesis [53, 54]. All evidence (strong and weak) will be synthesized into the framework using ecological triangulation as the narrative approach [44, 55].

Ecological triangulation allows identifying the interconnected relationships between the elements of the framework beyond the two-dimensional [44, 55]. The approach will highlight mutually interdependent relationships between team structure, team process, health outcomes, and environments. This approach also allows incorporating a discussion of the type of evidence (qualitative or quantitative) and the possible discrepancies in conclusions. Our Knowledge User Advisory Group has identified the resultant types of statements as most useful. An ecological statement might take the form: “*In this rural area, where the majority of the population has chronic care needs, a salaried team led by a nurse practitioner best supports access to comprehensive care.*”

The analytical framework described under “data extraction and analysis” will be relied upon in the creation of the decision support tool (Additional file 4: Table S1). The tool will report on the content of the evidence, and in addition, rate the evidential support of associations between particular team structure characteristics, and team processes or health system outcomes. The evidence rating is as follows:

- Strong evidence of effect—more than one study with a MMAT rating of ****
- Moderate evidence of effect—one or more studies with a MMAT rating of ***
- Limited evidence of effect—one of more studies with a MMAT rating of ** or less
- Conflicting evidence—inconsistent finding across studies, with MMAT ratings of *** or more
- No evidence—no studies, or conflicting findings with MMAT ratings of **or less

The ecological triangulation approach also allows for the discussion of the quality of the evidence on the basis of which specific conclusions drawn. For example, the above statement can be supplemented with “There is strong evidence to support this claim as per MMAT rating, and the majority of supporting studies are qualitative.”

#### Knowledge translation

Our project team takes an integrated knowledge translation approach. Our Knowledge User Advisory Group has been and will be involved directly in the development of the research objectives, the selection of inclusion/exclusion criteria, the discussion of the a priori framework for analysis, and the building of the reference guide and report. The Group has been consulted on the development and refinement of inclusion and exclusion criteria. The integrated knowledge translation approach allows for ongoing formative evaluation of the project, where knowledge users have the opportunity to monitor the process and ensure the usability of the resultant outcomes.

In addition to standard academic dissemination channels, the following outputs are proposed for dissemination to the knowledge user audiences.

1. An electronic decision support tool for decision makers at the policy and managerial levels based on the framework analysis. The tool will be a web-based database that serves as a quick reference guide (see Additional file 4: Table S1 for draft template).
2. A report for policy decision makers that identifies contextualized evidence behind optimal team design and the extent to which the evidence can be trusted, based on ecological triangulation.
3. A webinar for policy makers and managers to demonstrate the content and the use of the electronic reference guide.
4. A summary of the report on our website: http://www.primaryhealthcareteams.ca/

The decision support tool will be hosted on Dalhousie University servers, and the report along with a link to the database will be posted on our website (www.primaryhealthcareteams.ca). The knowledge users on our team will distribute the link to their networks. We will offer a webinar to demonstrate the use of the reference guide.

## Discussion

### Potential challenges and solutions

We anticipate and respond to three challenges in the proposed review. First is the challenge related to the reporting of qualitative research. Due to conventional space constraints imposed by journals, coupled with the lack of standardized reporting requirements, published qualitative studies are often not reported with sufficient breath and detail [56]. This limits our ability to judge the study’s quality. We will respond to this challenge by contacting study authors where necessary, to retrieve more complete reports on methods used in the qualitative studies.

Second, our proposed study is atypically broad. The breadth is dictated by the needs identified by the Knowledge User Advisory Group. We respond to this challenge by proposing a structured and well-matched process for retrieval, analysis, and synthesis developed specifically to address the diverse nature of research in this area.

Third, the explication of the search process for online non-academic studies presents a challenge. The online environment is less controlled than the academic library database system. We respond to this by creating an audit trail using the customized Grey Matters Tool and creating a tracking system for any digressions.

### Potential contribution

The resultant synthesis will be packaged into a web-based decision support tool. The integrated knowledge translation approach in the development of the decision tool will ensure its usability from the point of view of decision makers. We anticipate that the decision tool will be used in public payer health policy discussions focused on the design of policy-controlled elements of IDPC teams.

## Additional files

Additional file 1: PRISMA-P (Preferred Reporting Items for Systematic review and Meta-Analysis Protocols) 2015 checklist: recommended items to address in a systematic review protocol*. (DOC 84 kb)
Additional file 2: Comprehensive Medline Strategy. (DOC 75 kb)
Additional file 3: Structured search of non-academic literature. (DOC 69 kb)
Additional file 4: Table S1. Draft template for a decision support tool. (DOC 48 kb)

## Abbreviations

CADTH: Canadian Agency for Drugs and Technologies in Health
IDPC: Interdisciplinary primary care
MMAT: Mixed Methods Appraisal Tool
