# Supplementary material for: How best to structure interdisciplinary primary care teams: the study protocol for a systematic review with narrative framework synthesis
Source: Syst Rev. 2016 Oct 4;5:170. doi: 10.1186/s13643-016-0339-9 (PMC5050675; doi:10.1186/s13643-016-0339-9)
Supplement: Additional file 2: — Comprehensive Medline Strategy. (DOC 75 kb) [file 13643_2016_339_MOESM2_ESM.doc]

**Additional file 2 – Comprehensive Medline Strategy**

Provided is the Medline search strategy for the search involving interdisciplinary primary care teams and chronic conditions. The searches adapted for other databases (Embase, CINAHL, PAIS, Web of Science), and the supplementary search strategy involving interdisciplinary primary care teams and team structural characteristics are not provided here.

| [# ▲](http://ovidsp.tx.ovid.com/sp-3.18.0b/ovidweb.cgi?&S=JAGAFPNOLFDDECGANCJKODJCCHGGAA00&Sort+Sets=descending) | Searches | Results |
| --- | --- | --- |
| 1 | exp "Family Practice"/ or exp "Primary Health Care"/ or "Comprehensive Health Care".sh. or exp "General Practice"/ or exp "General Practitioners"/ or exp "Physicians, Family"/ or exp "Physicians, Primary Care"/ or exp "Primary Care Nursing"/ or ((primary or general or comprehensive or family) adj2 (nurse or nurses or nursing or doctor* or physician* or care or health or practitioner* or practice or healthcare or medicine)).tw. | 332679 |
| 2 | (((multidisciplin* or multi-disciplin* or interdisciplin* or inter-disciplin* or interprofession* or inter-profession* or collaborat*) adj3 (team* or group* or practice)) or ((doctor? or physician?) adj5 nurse? adj5 collaborat$)).tw. | 27834 |
| 3 | exp interprofessional relations/ | 58984 |
| 4 | (team* or group* or practice).tw. | 3259104 |
| 5 | 3 and 4 | 16480 |
| 6 | 2 or 5 | 41231 |
| 7 | 1 and 6 | 6652 |
| 8 | (("primary health" or "primary care" or "primary healthcare" or "general practice" or "family practice" or "family medicine") adj5 (team* or group* or collaborat* or interdisciplin* or "inter-disciplinary" or multidisciplin* or "multi-disciplinary" or interprofession* or "inter-professional")).tw. | 6577 |
| 9 | 7 or 8 | 12348 |
| 10 | "team climate inventory".tw. | 33 |
| 11 | 9 or 10 | 12372 |
| 12 | ((primary or general or comprehensive or family) adj1 (nurse or nurses or nursing or doctor* or physician* or care or health or practitioner* or practice or healthcare or medicine)).tw. | 209961 |
| 13 | exp "Family Practice"/ or exp "Primary Health Care"/ or "Comprehensive Health Care".sh. or exp "General Practice"/ or exp "General Practitioners"/ or exp "Physicians, Family"/ or exp "Physicians, Primary Care"/ or exp "Primary Care Nursing"/ | 193220 |
| 14 | 12 or 13 | 309721 |
| 15 | 3 and 14 | 7196 |
| 16 | (((multidisciplin* or multi-disciplin* or interdisciplin* or inter-disciplin* or interprofession* or inter-profession* or collaborat*) adj5 (team* or group* or practice)) or ((doctor? or physician?) adj5 nurse? adj5 collaborat$)).tw. | 31297 |
| 17 | 3 or 16 | 86658 |
| 18 | 14 and 17 | 10528 |
| 19 | 8 or 18 | 16018 |
| 20 | 10 or 19 | 16043 |
| 21 | exp diabetes mellitus/ | 341258 |
| 22 | diabet$.tw. | 463116 |
| 23 | IDDM.tw. | 6729 |
| 24 | NIDDM.tw. | 6815 |
| 25 | MODY.tw. | 930 |
| 26 | (non insulin$ depend$ or noninsulin$ depend$ or non insulin?depend$ or noninsulin?depend$).tw. | 11970 |
| 27 | (insulin$ depend$ or insulin?depend$).tw. | 28256 |
| 28 | (T1DM or T2DM).tw. | 11478 |
| 29 | 21 or 22 or 23 or 24 or 25 or 26 or 27 or 28 | 520092 |
| 30 | 20 and 29 | 930 |
| 31 | (asthma$ or wheez$ or bronchospas$ or (bronch$ adj3 spas$) or bronchoconstrict$ or (bronch$ adj3 constrict$)).mp. | 160752 |
| 32 | exp asthma/ or exp Bronchial Spasm/ | 114089 |
| 33 | ((bronch$ adj3 constrict$) or ((bronchial$ or respiratory or airway$ or lung$) adj3 (hypersensitiv$ or hyperreactiv$ or allerg$ or insufficiency))).mp. | 58631 |
| 34 | exp Bronchoconstriction/ or Bronchial Hyperreactivity/ or Respiratory Hypersensitivity/ | 18789 |
| 35 | 31 or 32 or 33 or 34 | 200497 |
| 36 | 20 and 35 | 234 |
| 37 | exp Myocardial Ischemia/ or Heart Diseases/ or exp Myocardial Revascularization/ or Cardiovascular Diseases/ | 561500 |
| 38 | (angina or cardiac).tw. | 507820 |
| 39 | exp Cardiac Surgical Procedures/ or exp Heart Function Tests/ | 608760 |
| 40 | ((heart or cardiac or myocardial or coronary) adj3 (disease* or infarct* or bypass* or ischemi* or failure*)).tw. | 501865 |
| 41 | cardiovascular.mp. | 428634 |
| 42 | exp heart failure/ | 95465 |
| 43 | ((heart or cardiac or myocardial or coronary) adj3 (disease* or infarct* or bypass* or ischemi* or failure* or attack*)).tw. | 504735 |
| 44 | exp myocardial infarction/ | 153027 |
| 45 | exp hypertension/ | 224497 |
| 46 | hypertens$.tw. | 346186 |
| 47 | exp blood pressure/ | 260545 |
| 48 | (blood pressure or bloodpressure).tw. | 238691 |
| 49 | 37 or 38 or 39 or 40 or 41 or 42 or 43 or 44 or 45 or 46 or 47 or 48 | 1930201 |
| 50 | 20 and 49 | 1216 |
| 51 | ((persistent or long* term or ongoing or degenerative or chronic) adj3 (disease* or ill* or condition* or insufficienc* or disorder* or care)).tw. | 297924 |
| 52 | long term care/ | 22780 |
| 53 | 51 or 52 | 311898 |
| 54 | 20 and 53 | 1403 |
| 55 | 29 or 35 or 49 or 53 | 2704679 |
| 56 | 20 and 55 | 2823 |
