# Supplementary material for: How best to structure interdisciplinary primary care teams: the study protocol for a systematic review with narrative framework synthesis
Source: Syst Rev. 2016 Oct 4;5:170. doi: 10.1186/s13643-016-0339-9 (PMC5050675; doi:10.1186/s13643-016-0339-9)
Supplement: Additional file 3: — Structured search of non-academic literature. (DOC 69 kb) [file 13643_2016_339_MOESM3_ESM.doc]

**Additional file 3 – Structured search of non-academic literature**

The GreyMatters Tool is adapted for our particular context. This strategy will be emergent during the search.

|  | Grey Matters Checkbox | | | |
| --- | --- | --- | --- | --- |
| Source | Searched; results found | Searched; nothing found | Results of peripheral interest | Not searched; not relevant |
| **Grey Matters - Health Economics – Canadian sites** |  |  |  |  |
| Hospital for Sick Children (Toronto) |  |  |  |  |
| Institute of Health Economics |  |  |  |  |
| McMaster University, Centre for Health Economics and Policy Analysis |  |  |  |  |
| Ontario Ministry of Health and Long-Term Care |  |  |  |  |
| Public Health Agency of Canada |  |  |  |  |
| Toronto Health Economics and Technology Assessment Collarboative |  |  |  |  |
|  |  |  |  |  |
| **Grey Matters - Health Economics – International sites** |  |  |  |  |
| Agency for Healthcare Research and Quality |  |  |  |  |
| Australian Government Department of Health and Ageing |  |  |  |  |
| Federal Reserve Bank of St. Louis, Economic Research Division |  |  |  |  |
| International Society for Pharmacoeconomics and Outcomes Research |  |  |  |  |
| John Wiley & Sons, Inc. Health Economic Evaluations Database |  |  |  |  |
| National Centre for Pharmacoeconomics, Ireland |  |  |  |  |
| NHS EED, economic evaluations of health care interventions |  |  |  |  |
|  |  |  |  |  |
| **Our Added Websites** |  |  |  |  |
| Alberta Health Services |  |  |  |  |
| Alberta Innovates Health Solutions |  |  |  |  |
| Atlantic Health Promotion Research Centre |  |  |  |  |
| Canadian Foundation for Health Care Improvement |  |  |  |  |
| Canadian Health Policy Institute |  |  |  |  |
| Canadian Institute for Health Information |  |  |  |  |
| Canadian Medical Association |  |  |  |  |
| Canadian Nurses Association |  |  |  |  |
| Canadian Research Network for Care in the Community |  |  |  |  |
| The Change Foundation |  |  |  |  |
| Child and Family Research Institute |  |  |  |  |
| The Conference Board of Canada |  |  |  |  |
| Fraser Institute |  |  |  |  |
| Health System Performance Research Network |  |  |  |  |
| Institute for Clinical Evaluative Science |  |  |  |  |
| Institute for Research on Public Policy |  |  |  |  |
| McGill University, Institute for Health and Social Policy |  |  |  |  |
| National Collaborating Centre for Health Public Policy |  |  |  |  |
| New Brunswick Ministry of Health Epidemiological Services |  |  |  |  |
| Newfoundland & Labrador Centre for Applied Health Research |  |  |  |  |
| Nova Scotia Department of Health and Wellness |  |  |  |  |
| Ontario Medical Association |  |  |  |  |
| Ontario Ministry of Health and Long-term Care |  |  |  |  |
| Quebec Population Health Research Network |  |  |  |  |
| Registered Nurses’ Association of Ontario |  |  |  |  |
| Saskatchewan Health Research Foundation |  |  |  |  |
| Tommy Douglas Research Institute |  |  |  |  |
| University of British Columbia Centre for Health Services and Policy Research |  |  |  |  |
| University of Calgary, O’Brien Institute for Public Health |  |  |  |  |
| University of Calgary, Snyder Institute for Chronic Diseases |  |  |  |  |
| University of Manitoba, Manitoba Centre for Health Policy |  |  |  |  |
| University of Ottawa, Institute of Population Health |  |  |  |  |
| University of Toronto, Institute of Health Policy, Management and Evaluation |  |  |  |  |
| Wellesley Institute |  |  |  |  |
