# Supplementary material for: How best to structure interdisciplinary primary care teams: the study protocol for a systematic review with narrative framework synthesis
Source: Syst Rev. 2016 Oct 4;5:170. doi: 10.1186/s13643-016-0339-9 (PMC5050675; doi:10.1186/s13643-016-0339-9)
Supplement: Additional file 4: Table S1. — Draft template for a decision support tool. (DOC 48 kb) [file 13643_2016_339_MOESM4_ESM.doc]

**Additional file 4: Table S1 – Draft template for a decision support tool**

The decision tool will have three layers, each providing more detail about a subset of information contained in the previous layer. The goal of the first layer is to provide a broad overview over the state of knowledge. The goal of the second layer is to provide a more focused overview of linkages between specific structural elements and selected outcomes. The goal of the third layers is to describe in more detail the extant knowledge of the connection between one structural elements and a selected outcome.

**Layer 1 – Broad overview**

|  | Policies and Procedures | Team Composition | Provider Remuneration | Team Funding | Team Governance |
| --- | --- | --- | --- | --- | --- |
| Team Process | **A1** | **A2** | **A3** | **A4** | **A5** |
| Health Services Process | **B1** | **B2** | **B3** | **B4** | **B5** |
| Diabetes Care | **C1** | **C2** | **C3** | **C4** | **C5** |
| Hypertension Care | **D1** | **D2** | **D3** | **D4** | **D5** |
| Asthma Care | **E1** | **E2** | **E3** | **E4** | **E5** |
| Ischemic Heart Disease Care | **F1** | **F2** | **F3** | **F4** | **F5** |
| Other Chronic Disease Mgmt  Outcomes | **G1** | **G2** | **G3** | **G4** | **G5** |

Cells will be coded for the strength of evidence as described. Some cells may be empty, indicating an absence of studies. The results of the synthesis are not yet available (this is the study protocol). As an example, cell C1 might read “*Moderate evidence of clear policies and procedures improving care for diabetes*”.

**Layer 2 – Focused overview**

Selected cells from the first layer are expanded here. The electronic tool allows the user to click on any cell of the first layer and enter the corresponding second layer.

A1

| Team Process | Policies and Procedures | | |
| --- | --- | --- | --- |
|  | Clear common goals | Roles and responsibilities | Process Guidelines |
| Perception of team functioning | **A1a1** | **A1a2** | **A1a3** |
| Measured team functioning | **A1b1** | **A1b2** | **A1b3** |

**B**2

| Health Services Process | Team composition | | |
| --- | --- | --- | --- |
|  | Add a nurse | Add a pharmacist | Add a dietician |
| Access to Care | **B2a1** | **B2a2** | **B2a3** |
| Care Comprehensiveness | **B2b1** | **B2b2** | **B2b3** |
| Satisfaction with care | **B2c1** | **B2c2** | **B2c3** |

**C3**

| Diabetes Care | Remuneration of Providers | | |
| --- | --- | --- | --- |
|  | FFS to physician | Salaries to all | Other mixed remuneration |
| Diabetes Care – Process and Service Delivery | **C3a1** | **C3a2** | **C3a3** |
| Diabetes Care – Patient Outcomes | **C3b1** | **C3b2** | **C3b3** |

The results of the synthesis are not yet available (this is the study protocol). As an example, C3a2 might read: “When all providers receive a salary, the cycle of care is more likely to be completed. Strong evidence”.

**Layer 3 – Narrative ecological statements**

The user can click on the result described in C3a2 and will be able to view a narrative statement describing the synthesis of the evidence linking salaries to the cycle of diabetes care. The synthesis contextualizes the evidence into ecological statements that identify conditions in which the stated relationship between salaries and diabetes care is most likely to hold.

In addition, the user will be able to click on the general category “remuneration of providers” to view a narrative ecological synthesis of the extant knowledge around the effects of provider remuneration on all aspects of care under investigation.
